# A PLAN to address the Parkinson pandemic

**DOI:** 10.1177/1877718X251378115

**Published:** 2025-10-21

**Authors:** E Ray Dorsey, Michael S Okun, Bastiaan R Bloem

**Affiliations:** 1 Atria Health and Research Institute, New York, NY, USA; 2 Center for the Brain & the Environment, Atria Research and Global Health Institute, New York, NY, USA; 3 Norman Fixel Institute for Neurological Diseases, Department of Neurology, University of Florida, Gainesville, FL, USA; 4 Donders Institute for Brain, Cognition and Behavior, Department of Neurology, Radboud University Medical Center, Nijmegen, The Netherlands

**Keywords:** Parkinson disease, epidemiology, environment, pesticides, trichloroethylene, solvents, air pollution, patient advocacy, delivery of health care, therapeutics

## Abstract

The Parkinson pandemic continues to spread. Almost 12 million individuals now have the disease, nearly double the estimate from just six years ago. Its human-made nature is also increasingly clear as more studies tie environmental toxicants to the disease. Chief among these are certain pesticides, the dry-cleaning chemicals trichloroethylene and perchloroethylene, and air pollution. An etiological role for these toxicants—inhaled or ingested—is also consistent with the emerging brain- and body-first models of Parkinson's disease.

To address the pandemic will require a “PLAN” that (1) Prevents the disease; (2) Learns why it starts; (3) Amplifies the voices of persons with the disease and their caregivers; and (4) Navigates the frontier of new treatments. Reducing or eliminating toxicants will help slow its rise. Learning why the disease begins will require investigating exposures, interactions of the environment with genes, and modifiers. Amplifying the voices of those affected can raise awareness, improve care, and change the disease's course. Vastly expanding the scale and scope of research funding will accelerate efforts to prevent the disease and find more effective therapies. If successfully implemented, such a plan will translate to bold “**0-10-100”** goals by 2035. The goals include a **0%** rise in the global incidence of Parkinson's, a **10-fold** increase in research funding and in the proportion devoted toward prevention, and **100%** of individuals having access to levodopa and receiving appropriate care. The results will lay the foundation for even greater ambitions, including the fall of Parkinson's disease.

## Plain language summary

Parkinson's is one of the world's fastest growing brain diseases, and most of it is preventable. Adjusted for age, the prevalence of Parkinson's has increased 60% over the last thirty years. Chemicals in our food, water, and air are helping fuel its rise. Many of these are inhaled (come in through the nose) or ingested (enter through the gut). To address the Parkinson pandemic, we propose a “PLAN” to (1) Prevent the disease; (2) Learn why it starts; (3) Amplify the voices of those affected; and (4) Navigate the frontier of new treatments. By doing so, we can reach bold goals by 2035. These include a **0%** rise in the number of new cases of the disease (adjusted for age), a **10-fold** increase in funding for Parkinson's research and the proportion devoted toward prevention, and **100%** of individuals having access to levodopa and appropriate care. By doing so, we will set the stage for the fall of Parkinson's disease.

“It takes as much as energy to wish as it does to plan.”

– Eleanor Roosevelt

Forty years ago, a new disease with an unknown cause threatened to spread, disable, and kill millions. In response, victims were blamed for their own illness, many doctors refused to offer care, some of the world's most prestigious hospitals closed their doors, and governments looked the other way.^1,2^ Into this vacuum, a group of brave individuals adopted a slogan of “Silence = Death.” Together they changed the hearts and minds of citizens, confronted corporate interests, and challenged political leaders. The result for HIV/AIDS has been nothing short of miraculous.

While much more work remains, the pandemic of HIV is receding, and the global incidence is declining.^3^ The feared infection could, with continued efforts, gradually disappear—an outcome that was unfathomable even a decade ago. A primary focus on prevention, a substantial investment in research, a commitment to care for all, and the development of novel treatments have changed the disease's course. Today, HIV is preventable with a condom, NIH funding for the disease exceeds $3 billion annually, and the vast majority of affected individuals are diagnosed and receive appropriate treatment. All this progress has been made with only a small handful of “cures.”^4^

By contrast, the pandemic of Parkinson's disease (PD) is growing and spreading fast. Its major environmental causes are under investigated,^5^ prevention is rarely considered, and large numbers receive their diagnosis far too late or go undiagnosed altogether. Most people do not receive appropriate treatment, and therapeutic progress has been slow.^6^ In short, we are failing the Parkinson's community.

Here, we review the recent evidence of the global rise of PD, highlight its increasingly clear human-made nature, and outline a “PLAN” to change its course. The plan includes **P**reventing the disease, **L**earning why it starts, **A**mplifying the voices of those affected, and **N**avigating the frontier of new therapies. If implemented, we can slow and perhaps even stop the rise of Parkinson's in the next decade, vastly increase funding for the disease, and improve access to care and treatment.

## The rise of Parkinson's disease

In his 1817 “Essay on the Shaking Palsy,” Dr James Parkinson described six individuals with a novel disease that – in his words – had hitherto “escaped particular notice.”^7^ While ancient Chinese, Indian, and Egyptian descriptions of the disease (or related ones) exist,^8,9^ the disease was likely rare prior to the nineteenth century.

Two centuries after Parkinson's description of six individuals with the disease, the Global Burden of Disease study estimated that over six million individuals were affected.^10^ This rapid rise, seen in almost every part of the world, led us to characterize the phenomenon as the “Parkinson's pandemic.”^11,12^

According to researchers from the National Institute of Allergy and Infectious Diseases, the “only 1 invariable common denominator” of a pandemic seems to be widespread geographic extension, which PD clearly meets.^13^ Other proposed key features of a pandemic are disease movement, high attacks and explosiveness, minimal population immunity, novelty, severity, infectiousness, and contagiousness. PD also satisfies many of these as the disease is most common in industrialized nations, like Canada and the U.S, least common in less industrialized nations like those in sub-Saharan Africa, and rising greatest in countries that have undergone rapid industrialization (e.g., India and China).^14^ The growth of PD has been explosive from an estimated 2.6 million in 1990 to 11.8 million in 2021.^14,15^ No one is known to be immune to PD. While ancient descriptions exist, the disease was novel to the eyes of 61-year-old Dr James Parkinson, and the vast majority of the individuals affected by the disease have occurred in the past century. The disease is clearly severe, disabling, and deadly (now the 14^th^ leading cause of death in the U.S.).^6,16^ While most pandemics have classically been infectious and thus transmissible or contagious, some are asking whether we are now confronting pandemics of chronic diseases.^16^

The epidemiologist Abdel Omran was interested in what were the drivers of population growth. In 1971, he wrote a landmark paper in which he argued that humans have transitioned from a period or receding (infectious) pandemics to an era of degenerative man-made diseases.^17^ The “vectors” of these chronic conditions, which are now the principal sources of disability and death globally, are not bacteria or viruses but include cigarettes, ultra-processed foods, and environmental changes.^16^ As just one example, lung cancer has gone from a “once-in-a-life oddity” at the turn of the twentieth century to the most common cause of cancer in the world today.^18^

PD is not the only chronic condition that has been labelled a “pandemic.” Because of their widespread prevalence, migration to areas adopting Western diets, rapid rise, absence of apparent immunity, novelty, and severity, many have referred to the rise of obesity and type II diabetes as pandemics.^13,19,20^ The main purpose of characterizing a disease as an epidemic or pandemic is to highlight a “danger to the public and a very large number of victims.”^16,21^ PD is certainly that.

**Figure 1.**
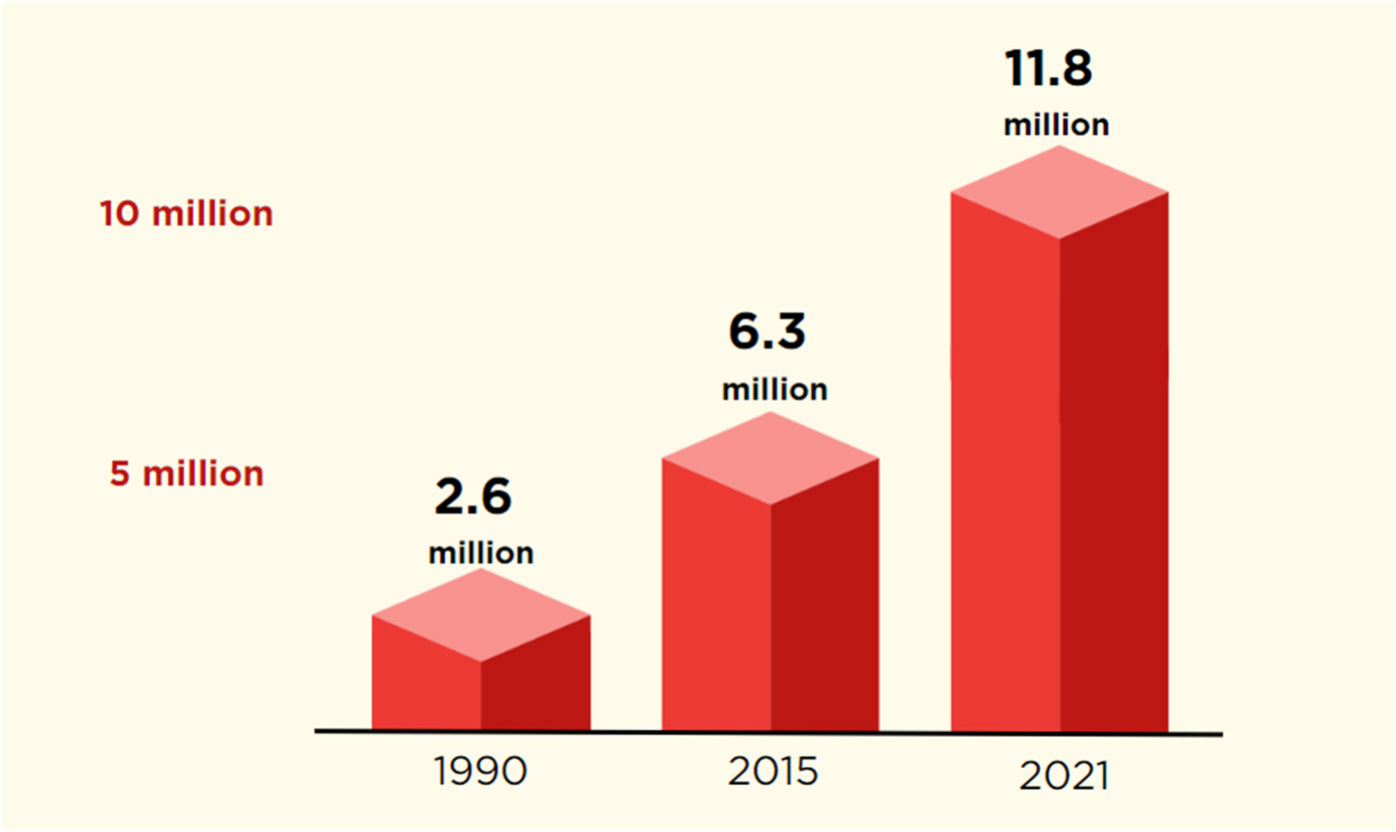
Estimated prevalence of Parkinson's disease worldwide per Global Burden of Disease Study.^6^

While some have contested the notion of the “Parkinson pandemic,”^22^ the global rise of PD has only continued. According to the Global Burden of Disease study, 6.3 million individuals had PD in 2015, more than twice its estimate (2.6 million) in 1990.^10^ In the process, it identified Parkinson's as the world's fastest growing neurological disorder.^23^ In 2024, the same group reported that 11.8 million individuals had PD in 2021 (Figure 1),^15^ nearly double its calculation from just six years earlier, and greater than what we had projected for 2035.^12^ The evidence supporting the rise in PD is not limited to the Global Burden of Disease study. For example, Willis and colleagues recently estimated that the incidence of PD in the U.S. in 2012 may be 90,000, or 50% higher than previous studies.^24^

Aging alone is insufficient to explain the rise. The change in prevalence from 1990 to 2021 was 60.7% *after* adjusting for age. By contrast, the age-adjusted prevalence of Alzheimer's disease increased by only 3.2% during that period, while motor neuron disease (amyotrophic lateral sclerosis) decreased by 1.3%.^15^

Improved diagnosis is also not a likely explanation. The age-adjusted prevalence has decreased for diseases which our diagnostic ability has unquestionably improved, including multiple sclerosis (–0.3%) and stroke (–8.5%),^15^ diseases for which our diagnostic ability has unquestionably improved.

## Its man-made nature

A genetics revolution has vastly increased our knowledge of the underpinnings of many diseases, including Parkinson's. Since 1997, at least seven underlying genetic “causes” of Parkinson's have been identified along with numerous genetic risk factors for the disease.^25,26^ These advances have enhanced our understanding of its pathophysiology, including Parkinson's characteristic mitochondrial and lysosomal dysfunction, enabled genetic testing, and spurred the development of gene-directed therapies.

**Figure 2.**
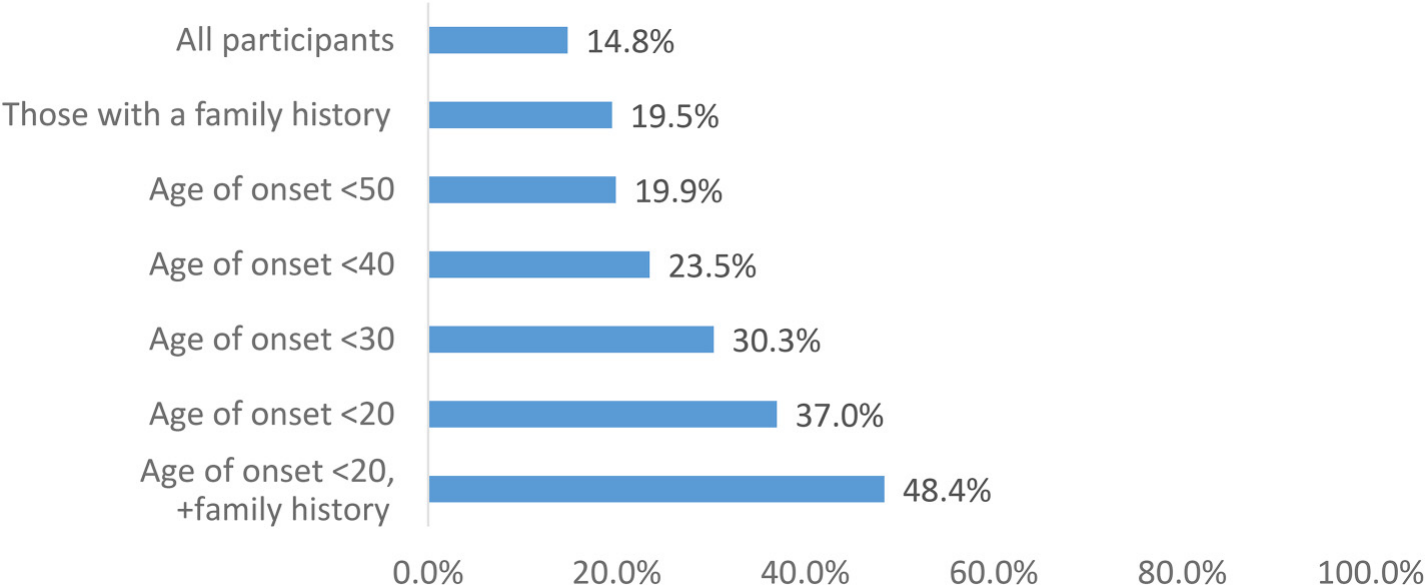
The proportion of individuals with Parkinson's with a “PD-relevant genetic test” in the Rostock International Parkinson’s Disease Study.^28^

However, these genetic factors do not explain why most individuals with PD have the disease. In China, 5% of individuals with sporadic early-onset PD have a genetic risk factor.^27^ In North America, only 13% carry one of the seven most common genes for the disease.^25^ Similarly, just 15% of 12,580 individuals with PD across sixteen countries in Europe, the Middle East, and North and South America carry a genetic marker.^28^ Even among individuals with an age of onset before 50, only 20% have a genetic explanation. For those with an age of onset less than 20 years old and a family history of the disease, the majority do not carry a PD-relevant genetic mutation (Figure 2).^28^

Of course, these studies only assess known genetic factors, and many common variants, likely with small effects, remain to be identified.^29^ However, they all indicate that the vast majority of individuals with PD at almost every age in much of the world do not have a primary genetic explanation for their disease.

That said, a genetic predisposition may make individuals more susceptible to the effects of environmental toxicants. Indeed, when genetic risk factors for PD are introduced into experimental animals, they are more likely to develop signs of neurotoxicity following exposure to pesticides. Such a gene-environment interaction could explain, in part, why certain farmers who work with pesticides develop PD while most do not. The latter may lack an underlying genetic susceptibility or are protected because of genetic differences, such as a polymorphism in a gene that encodes for detoxification, or are just fortunate.

A genetic predisposition is by itself unlikely to be a sufficient explanation for developing PD because many of the known PD mutations have been around for millennia, yet the disease was rare prior to the nineteenth century.^30^ The relevance of these genetic differences may have only manifested after we clouded our air with particulate matter during the Industrial Revolution and polluted our food and water with excessive synthetic pesticides following World War II.

The principal explanation for PD is the environment.^31,32^ Numerous environmental and behavioral factors have been linked to a higher or lower risk of PD, including diet and nutritional factors, head trauma, infections, medications, metals, naturally occurring toxins, exercise, and smoking.^33,34^ Here, we focus on pesticides, the industrial solvents trichloroethylene (TCE) and perchloroethylene (PCE), and air pollution—toxicants that are either common, have been associated with large effect sizes, or both, and are addressable.

Over the past six years, the emerging evidence^35^ of the environmental causes of the Parkinson's pandemic has soared. Perhaps the greatest contribution has come from an unlikely source, a pesticide manufacturer. A 2022 exposé in *The Guardian* reviewed legal documents from a manufacturer of paraquat as part of lawsuits from farmers who had worked with the chemical and subsequently developed PD.^36^ According to the piece, experiments by a manufacturer itself showed that high paraquat exposure in three different mammalian species, mice, rats, and rabbit, produced the symptoms of Parkinson's, including tremors. The studies were conducted in the 1960s, a generation before academic researchers had reached a similar conclusion.^37^

In addition, the reporters found that the company withheld important information from regulators, set up a team to discredit the work of scientists investigating paraquat, knew that long-term spraying could injure the central nervous system, and were concerned that legal liability for paraquat could mirror that of asbestos noting that “Parkinson's can go on for decades.”^36^

The epidemiological evidence linking paraquat to Parkinson's has also continued to mount. In 2024, Paul and colleagues found that risk of PD among individuals who lived or worked near where paraquat was sprayed in California doubled.^38^ An accompanying editorial argued that based on the results, industry must now have the burden of proving that paraquat is unequivocally safe, rather than demanding that scientists demonstrate that the weed killer causes PD.^39^ Like second-hand smoke, the risk of these chemicals extends far beyond those who use them, and inhalation may be a potent route of exposure.^40^ Recent studies have also bolstered the evidence linking other pesticides to PD, including fat-soluble organochlorines.^41–43^

In addition to certain pesticides, the dry-cleaning chemicals trichloroethylene (TCE) and perchloroethylene (PCE) are emerging as important risk factors, if not causes, of PD. A landmark study by Goldman and colleagues examined members of the military who served at the U.S. Marine base Camp Lejeune. The drinking water there was contaminated by TCE, PCE, and other chemicals from a local dry cleaner and additional sources.^44^ They found that the prevalence of PD among service members (average age 20, average duration at the base 25 months) at Camp Lejeune was 70% higher than those who served at a less polluted base in California.

The chemicals, which are still widely used today, have contaminated the groundwater of countries around the world, including in the Americas, Africa, Asia, Australia, and Europe. In addition to posing a risk from ingestion, these volatile chemicals can, like radon, evaporate from contaminated soil and water and enter homes, schools, and work places, usually undetected.^45,46^

Laboratory studies, many by De Miranda and colleagues, have supported the epidemiological findings. TCE, when ingested, alters the gut microbiome of rats in ways similar to that observed in humans with PD.^47^ In addition, inhaled TCE in rodents appears to cause “highly potent dopaminergic neurodegeneration and recapitulates some of the observed neuropathology associated with PD.”^48^ TCE, like *LRRK2* mutations, also increases the activity of LRRK2 kinase,^49^ and inhibition of LRRK2 activity protects against TCE-induced toxicity.^50^

Outdoor air pollution, which darkened the skies of nineteenth century London, is an increasingly recognized risk factor for PD.^51–53^ Several recent studies have tied particulate matter – tiny pieces of dirt and soot suspended in the air – to PD.^52,54,55^ In the U.S., levels of particulate matter above the median were associated with a 56% increased risk of incident PD compared to those experiencing the lowest level of air pollution.^56^ A large European study also found that higher levels of particulate matter were associated with increased PD mortality.^57^ As with certain pesticides and TCE, these epidemiological findings have been supported by laboratory studies.^58^ For example, particulate matter smaller than 2.5 microns “triggers the fibrillization of [alpha-synuclein] and promotes the formation of [alpha-synuclein] fibrils” and neurotoxicity in mice.^59^ Studies of air pollution and PD, however, have been inconsistent,^60,61^ and additional investigations of these toxicants and others are needed.

## A new model for Parkinson's disease

The means by which these toxicants and other toxins (e.g., heavy metals) may contribute to PD is beginning to emerge. In 2019, Borghammer and Van Den Berge postulated two dominant models of Lewy body disorders.^62^ In the brain-first model of the disease, pathology begins in the olfactory bulb and then spreads primarily within one hemisphere (99% of neuronal connections are ipsilateral) to the substantia nigra via the amygdala and then down the brain stem and into the hemispheres. The result is an asymmetric disorder with early motor features and only later appearance of autonomic, sleep, and cognitive dysfunction.

By contrast, in the body-first model of Lewy body disorders, alpha-synuclein pathology begins in the gut and then spreads bilaterally via sympathetic and parasympathetic fibers (the gut receives bilateral innervation) to the spinal cord, heart, and brain stem before later reaching the substantia nigra and then the cerebral hemispheres. The result is early autonomic dysfunction (e.g., constipation), sleep disturbance (e.g., REM sleep behavioral disorder), more symmetric parkinsonism, and earlier cognitive impairment.

Inhaled and ingested environmental toxicants, including pesticides, solvents, and air pollution, fit well with this model as they come into contact with both the nose and the gut.^63^ Inhaled toxicants may predispose to a brain-first model of disease, while ingested ones could lead to a body-first model.^64^ While promising, the paradigm does not explain all cases (additional sub-types may be present) or address the unique aspects of PD for every individual. The models also require additional investigation and refinement, as some studies have not supported them.^65^

## A “PLAN” to address the Parkinson's pandemic

Perhaps the biggest recent step toward addressing the Parkinson's pandemic was the recognition of the need to do so. In 2022, the World Health Organization identified PD as a “global priority” and identified six areas to address the disease including disease burden; advocacy and awareness; prevention and risk reduction; diagnosis, treatment, and care; caregiver support; and research.^66^ In 2024, the U.S. Congress passed and President Biden signed the National Plan to End Parkinson's Act. Named after a physician who died from PD and a Congresswoman who developed progressive supranuclear palsy, the law requires the federal government to develop a plan to prevent and end the disease.^67^ While no funding is tied to the law, the hope is that it will set the stage for increased public awareness, greater investment, and efforts to improve prevention, care, and treatment.^68^ Here we provide a high-level “PLAN” that seeks to (1) **P**revent PD; (2) **L**earn why the disease starts, spreads, and progresses; (3) **A**mplify the voices of those affected; and (4) **N**avigate the frontier of new treatments.^69^

### Prevent

The first step to addressing any crisis is to prevent it. In response to the rising global burden of dementia, the *Lancet* Commission has produced reports highlighting the potentially preventable nature of dementia.^70^ In 2024, its report indicated that 45% of cases of dementia, which has a far greater heritability than PD, are potentially preventable.^71^ To our knowledge, no similar reports or global actions have been taken to address PD. Rather, we are only fueling the rise of PD through the continued and expanding use of chemicals linked to the disease. The result is unnecessary, unspoken, and unmeasured suffering. To prevent PD, we must measure the disease, ban toxic chemicals, and adopt the precautionary principle.

1. *Measure the disease* – What gets measured gets managed.^72^ And right now we are not measuring PD well, and the disease is out of control. We don’t know how many people globally or in any part of the world actually have it. Nor do we know how many individuals developed or were diagnosed with the condition last year. Prevalence and incidence are two important epidemiological measures, but we lack high quality data on both.^66^ The dearth of high-quality data, which is necessary to measure the disease, evaluate its causes, and track progress, is a symptom of our lack of commitment to addressing the Parkinson's pandemic.
  Current prevalence estimates are often based on models, claims data, or analyses from multiple studies, the values of which can vary by a factor of two or more.^24^ There is also a grave shortage of high quality data on PD incidence, which leads to debates on trends in the disease.^22^ A precious few studies have examined the incidence over time, but these valuable studies in Rochester, Minnesota,^73^ and Rotterdam, the Netherlands,^74^ for example, are unlikely to generalize to other parts of the state, country, and world where the environment (and genetics) likely differ.
2. *Ban toxic chemicals* – Removing or reducing toxic chemicals, such as lead, chlorofluorocarbons, and DDT, has improved health and the environment without damaging the economy. The same will likely be true for pesticides, such as paraquat and chlorpyrifos, and dry cleaning and degreasing chemicals like TCE and PCE. Some parts of the world already function without them, but in others their use (e.g., paraquat in the U.S. and TCE in China) is only increasing.^75^ Some of these chemicals are sixty years old (e.g., paraquat) and others (e.g., TCE) are a century. Chemists can surely develop safer alternatives – some are already promoted^76,77^ – and help prevent PD without a loss of productivity.^78^ And the price of clothes that don’t shrink should not be PD.
3. *Adopt the Precautionary Principle* – For sixty years, the U.S. Food and Drug Administration has required that sponsors of new drugs demonstrate their safety and efficacy before they enter the market. The same principle should apply to chemicals. Currently, the burden of proof is on scientists, regulators, and the public to demonstrate a chemical is unsafe and thus should be removed.
  Proving that any given chemical is a cause of PD is incredibly difficult. Randomized, controlled trials are not feasible or ethical. Exposure may precede diagnosis by decades (the prodromal phase may be as long as 20 to 30 years). Numerous factors can confound the results, including the effects of simultaneous or sequential exposures, diet, exercise, and genetics. The long latency period and lack of quantitative measures within an individual or the person's own environment can increase the risk of false negative findings. In the interim, potentially toxic chemicals continue to be used.
  Instead, the burden of determining a product's safety should fall on its producer. In addition, improved screening procedures, especially for the risk of neurodegeneration, are needed.^79^ And independent third parties should conduct these assessments.

### Learn

In most of neurology, one of the first steps in evaluating an individual with a neurological disorder (e.g., headache, seizure, stroke) is to determine why the disease developed. Only with this information can it be appropriately prevented and treated. However, for PD and many neurodegenerative disorders, this is rarely done. When it is, the results can be dramatic.

When Langston asked why a middle-aged man rapidly developed parkinsonism after using a synthetic heroin, he first identified its environmental cause, launched new animal models, and opened the door to prevention.^80^ When Polymeropoulos learned why a large Italian family was affected with PD, he first found its genetic cause, revolutionized our understanding of its pathology, and paved the way for new treatments.^81^ We need to shift our scientific thinking upstream to find the beginning, not the end, of the disease and learn why it starts.

1. *Recognize that there are multiple causes of Parkinson's* – Parkinson's disease is actually Parkinson's diseases.^82^ Like breast cancer, which can be due to chemicals, radiation, genetics, and other factors,^60^ Parkinson's has many different causes and likely sub-types.^83^ We now know that, for many, PD has its origins outside the brain.^84^ It is time that we study the entire bodies of affected individuals.^5^ This can be done through imaging^85^ or through pathological examination of bodies (and brains) after people have passed.^86^ There are likely clues in individuals’ guts and noses, not to mention their hearts, skin, lungs, kidneys, and other organs that might be affected by chemicals like TCE. If we methodically assess the whole body and map changes over time, we can assemble the pieces of the Parkinson's puzzle, identify all its true origins, and better understand its unique nature for affected individuals.^5^
2. *Assess the roles of nature and nurture* – Smoking causes lung cancer, but only about 10% of smokers get it.^87^ There must be other factors that determine who develops cancer and who does not. The same is true for Parkinson's. Not every farmer who sprays paraquat, not every Marine who drinks TCE, and not everyone who breathes in polluted air develops PD. We must learn why.
  Some of it is due to the exposure (dose, duration, route, and timing), but interactions with genes and other environmental factors, chance, and modifiers, such as stress, diet, and other diseases, are likely important, too. However, they are poorly understood. Most genetic causes of PD alone are also insufficient to produce the disease. Some – *LRRK2* mutations, for example – may have important interactions with TCE, while others, such as *GBA* mutations, have interactions with pesticides.^88^ Genetic differences in metabolism and detoxification are also likely important. Better understanding these relationships will pave the way toward more effective prevention and treatment strategies.

1. *Measure the chemicals within us* – We know that certain chemicals contribute to PD. We now need to measure them. We test adults for cholesterol to prevent heart disease and children for lead to avoid intellectual disabilities. Both efforts have had tremendous health benefits. For example, compared to the 1970s, the level of lead in children's blood today is 95% lower.^89^ Bans on lead in gasoline and paint have made us all smarter.^90,91^ We should use that intellect to expand testing for chemicals that are known to cause cancer and likely Parkinson's. We can measure fat-soluble toxicants like organochlorine pesticides and perhaps TCE in autopsy specimens and make comparisons between those with and without PD. For the living, we can evaluate workers in high-risk occupations (e.g., farmers), those who live or have lived near contaminated sites (e.g., Camp Lejeune; Newport Beach, California),^45^ and clusters of Parkinson's.^46,92^ In the 1990s, researchers in Italy tested the blood of the general population for common pollutants, including TCE and PCE. They discovered these chemicals in about three-quarters of the population.^93^ What would the results be today? In Italy? Canada? China? We should find out.

### Amplify

To change the course of PD will require amplifying the voices of those most directly affected. In HIV, silence equaled death. For PD, silence equals suffering. Nearly twelve million people are now living with Parkinson's globally. Many more are undiagnosed. Together, the majority of these individuals go without adequate care or treatment.^94,95^ Almost all need more help, and they need it now. We must substantially increase resources and amplify the voices of those affected—patients, caregivers, and families—to reduce everyone's burden. Here are three concrete steps that we should take.

1. *Enable all to receive levodopa* – Fifty years after the introduction of levodopa, many countries around the world lack access to the highly effective medication. According to the World Health Organization, only 37 of 110 countries globally have access to levodopa in clinics.^66^ And where available, it is often unaffordable. In low-income nations, it is not available at all.^66^
  We have failed to deliver a simple, safe, effective, low-cost tablet to millions of people with a treatable disease. This lack of access must end. Those with HIV receive far more expensive, complex medications for their condition. We should enable those with Parkinson's to receive appropriate treatment for theirs. To do so, we need to invest in building logistics to procure, deploy, track, and distribute PD medications throughout lower-income nations, and we should develop programs that increase the supply of dopamine by planting and processing the dopamine-rich plant *Mucuna pruriens*.^96,97^
2. *Make insurance coverage of telemedicine standard* – Telemedicine for Parkinson's only became a widespread reality during the Covid-19 pandemic. However, in the United States, the provision was a time-limited teaser. The coverage is still temporary, and many benefits are slated to expire in 2025. We must make telemedicine an option for all as it can help reach those who are most underserved or most disabled.^98,99^ No one should be left behind because of who they are or where they live.
3. *Double the number of centers of excellence* – Less than 10% of Americans with PD receive their care from a PD specialist.^94^ By contrast, about 20% receive their cancer diagnosis from a National Cancer Institute-designated Cancer Center.^100^ Part of the reason is that we have too few hubs of Parkinson's care. The U.S. alone has over 2000 stroke centers^101^ to care for the roughly 800,000 new strokes annually and the seven million Americans who have had one. By contrast, for the approximately 1.2 million Americans with PD, the U.S. has only around 60–70 centers of excellence.^102–104^ We propose that for every 10,000 persons with PD, a center of excellence be available. To meet this need in the U.S. alone would require a doubling of the number of centers to 100 and a commensurate rise in public and private funding.
  Better care results in better outcomes. Individuals with Parkinson's who see a neurologist are about 20% less likely to fracture a hip, to be placed in a skilled nursing facility, or to die prematurely compared to those who do not.^95^ Yet 40% of Americans do not see a neurologist of any kind within four years of diagnosis. In Europe, the first right endorsed by the European Parkinson's Disease Association Charter is care from a doctor with a special interest in Parkinson's, yet many do not receive such care.^105^ China has an estimated 3.6 to 5.1 million people with PD; however, the country only has about 150 specialists.^106,107^ Caring for the growing PD population will not only require additional new centers but also new care models.^108,109^ Some of these models could empower clinicians of all types including physicians (e.g., general neurologists, geriatricians, internists), therapists,^110^ and other clinicians (from social workers to nurse practitioners). In addition, novel approaches and technologies that extend the reach and scale (e.g., group visits, asynchronous communication or care) of clinicians are needed.^99^

### Navigate

Every six minutes we delay developing a new therapy, another diagnosis of PD in the U.S. is made.^24^ All of those with PD want and need new therapies. Some may be available in the near-term; some will take longer. All will require a substantial increase in investment. The road to better treatments will require us to change our approach, reject false notions, and explore new ones. We must learn from HIV/AIDS and cancer and begin to shift toward combination approaches where the sum of the improvement exceeds the individual benefit.^111^ The strategy of approaching cancer cells at different points in their cycle laid the groundwork for effective treatments and in some cases cures. In HIV the AIDS Clinical Trials Group 320 showed that a triple drug combination approach reduced HIV viral load and delayed disease progression more than use of individual drugs. Combination drugs helped transform HIV from a fatal disease into a chronic and manageable one.^112^ In PD, we identified that the combination of carbidopa plus levodopa^113^ improved the tolerability and effectiveness of levodopa for generations of patients. The two drugs were better together. Of course, for combinations to work at least one of the drugs must be effective on its own.

Another important area is the development of practical and affordable biomarkers that can diagnose and more accurately monitor disease progression. These biomarkers will help us to reduce the number of patients necessary to adequately power clinical trials and thus move therapies through the pipeline quicker. We should remain open-minded, adapt to new technologies and approaches, and consider combined biomarker approaches.

Genes and “omics” technologies may also uncover new drugs and targets that may be addressed by gene therapies, gene editing, regenerative medicine, or other novel methodologies. A large part of this focus should refine our techniques and delivery methods for gene editing, nanomedicine, neuromodulation, and neuroimmune approaches. We should invest more in artificial intelligence (AI) and must develop the rationale, ethics and implementation of its use. Researchers remain interested in the development of Parkinson's-specific fingerprints and using AI, for example, could help decode the microbiome and identify new therapeutic targets.

There are three frontiers to navigate in Parkinson's treatment. The first frontier can be deployed now, and this frontier has the greatest potential to help people in the near future. One important avenue encompasses expanding Parkinson's care, by making sure that many more affected individuals can have access to specialized healthcare professionals. At the least, this should involve a neurologist or geriatrician, supported by a Parkinson nurse, but preferably also a range of other critically important professionals such as a physiotherapist, speech-language therapist or occupational therapist.^114^ Other areas include improvements in non-invasive brain stimulation techniques, and refinements in pump treatment technology. A further area relates to the development and implementation of low-risk, yet effective behavioral therapies, such as exercise^115,116^ and, to a lesser extent, stress management.^116^ Considerably more work is needed to evaluate which nutritional interventions will be beneficial for persons with PD.^117^

Over the next five to ten years, the second frontier includes the potential development of new drugs, diets, stem cells, vaccines, and immunotherapies. These treatments could in some cases be designed to address both symptoms and slow disease progression. The third frontier – a decade or more out – encompasses testing of both conventional and novel therapies, such as regenerating circuits, gene therapy, gene editing, drugs with novel mechanisms of action, more effective combination therapies, and nanomedicine. A long-term plan should also include developing a new generation of researchers.

Here are three steps we can begin taking now.

1. *Dramatically increase funding for Parkinson's research* –In 2024, the estimated National Institutes of Health research funding for PD was $251 million, a fraction of what we invest in HIV/AIDS ($3.3 billion).^118^ Even after adding in the support from the US Department of Defense ($16 million)^119^ and other federal sources (e.g., the Department of Veterans Affairs), this amount of investment is simply insufficient.
  We propose an Operation Warp Speed for PD. We need to increase funding tenfold and reach $3 billion in federal support annually. This level of expenditure has had immeasurable benefits for preventing and treating HIV, which affects a similar number of Americans as PD.^120,121^ Millions have never been infected, and millions receive appropriate treatment because of that investment. With wise investment, the same can be realized for PD.
2. *Invest in novel approaches such as nanotechnologies* – Nanomedicine is one potential example of a technology that holds immense promise for both diagnosis and treatment of Parkinson's, however at this time it remains largely unexplored. Nanomaterials could be possibly applied to Parkinson's to improve our diagnostic imaging, and nanosensors could detect the presence of biomarkers in the blood and other body fluids. Nanomedicine may possibly improve drug delivery, reduce side effects, and enhance our ability to target specific brain regions. Nanomedicine has immense therapeutic potential for Parkinson's as it could more effectively penetrate the blood-brain barrier and enable delivery of nanoparticles, genes, and proteins.^122^
  To reduce harmful inflammation, we could, for example, evaluate loading nanoparticles with anti-inflammatory drugs. To rescue a brain damaged by a chemical, we could consider delivering nanoparticles to mitochondria to restore function. To fix damaged parts of the brain, we could deploy nanorobots, and nanovaccines to potentially prevent the spread of misfolded proteins.^123^
3. *Rethink regeneration* – Cell transplants and regenerative medicine may be a possible future option to improve memory, thinking, and learning. Previously many experts viewed regenerative medicine as closer to a cure. We think it is more reasonable to think of the near future of this therapy as symptomatic therapy targeted at medication-resistant symptoms. Using new targets, such as the basal forebrain, rich in acetylcholine, could provide a new avenue for regenerative medicine. Transplantation of acetylcholine-producing cells into brains of those with PD could improve memory and cognitive function.^124^ Similarly, approaches using gene therapy, CRISPR gene editing, and other new technologies should be embraced and funded. We should engage with bioengineers and other disciplines to develop new tools to approach circuit regeneration and repair.

## Bold goals

Plans need goals, and we can look to the HIV community for guidance. In 2013, UNAIDS sought to end the AIDS epidemic by 2030. To do so, they set three (90-90-90) targets for seven years in the future, 2020: 90% of those with HIV would be diagnosed, 90% of those would be treated, and 90% of them would have viral suppression.^125^ For PD, we can set 0-10-100 goals. By 2035, we can see a **0%** increase in the global incidence of PD; a **10**-fold increase in research funding for PD including a 10-fold increase in the proportion aimed at prevention; and **100%** of individuals with PD having access to levodopa.

## Goal 1: 0% rise in the global incidence of Parkinson's disease

By preventing PD, we can slow and halt its rise. PD has long prodromal period, measured in years to decades,^126^ which may represent a challenge to short-term changes, but reducing ongoing exposure may have near- and long-term benefits. We already see signs that this is possible. For example, use of pesticides (especially of the most hazardous ones) in Europe is either plateauing^127^ or declining. TCE use in Europe has decreased by 95% in the past dozen years.^128^ After having the world's worst air quality in the nineteenth century, Europe has seen emissions decrease by nearly 90% since its peak in 1980.^129^ Perhaps as a result, when adjusted for age, the prevalence of PD is increasing more slowly in western Europe (8.4%) for example, than in the world as a whole (21.7%).^14^ Potentially reflecting reductions in environmental toxicants, the incidence of PD in Germany^130^ is declining.^131^ In addition, the Rotterdam study, although with limitations and uncertainties, saw a 60% decrease in the incidence of PD in just two decades (1990 to 2011).^74^ The results suggesting that in at least some parts of the world, preventive measures such as the banning of paraquat are beginning to take effect,^27^ but the overall burden, even in the Netherlands, continues to rise.^28^

While PD may have peaked in some parts of the world, other parts may be at risk of an increasing incidence. In sub-Saharan Africa, pesticide use has tripled since 1990,^132^ and about 60% of its workforce is engaged in farming.^133^ Toxic pesticides that have been banned in many areas of the world are still being used in Africa where, combined with a young population, could set the stage for future generations of PD. The Middle East has some of the worst air quality in the world, and China, which has reduced its pesticide use and air pollution, accounts for half of the world's market of TCE.^45^

## Goal 2: 10-fold increase in research funding and in the percentage devoted to prevention

In the U.S., large increases in research funding in short time periods have precedence. It happened with HIV, and federal funding for Alzheimer's research recently increased seven-fold in only nine years.

**Figure 3.**
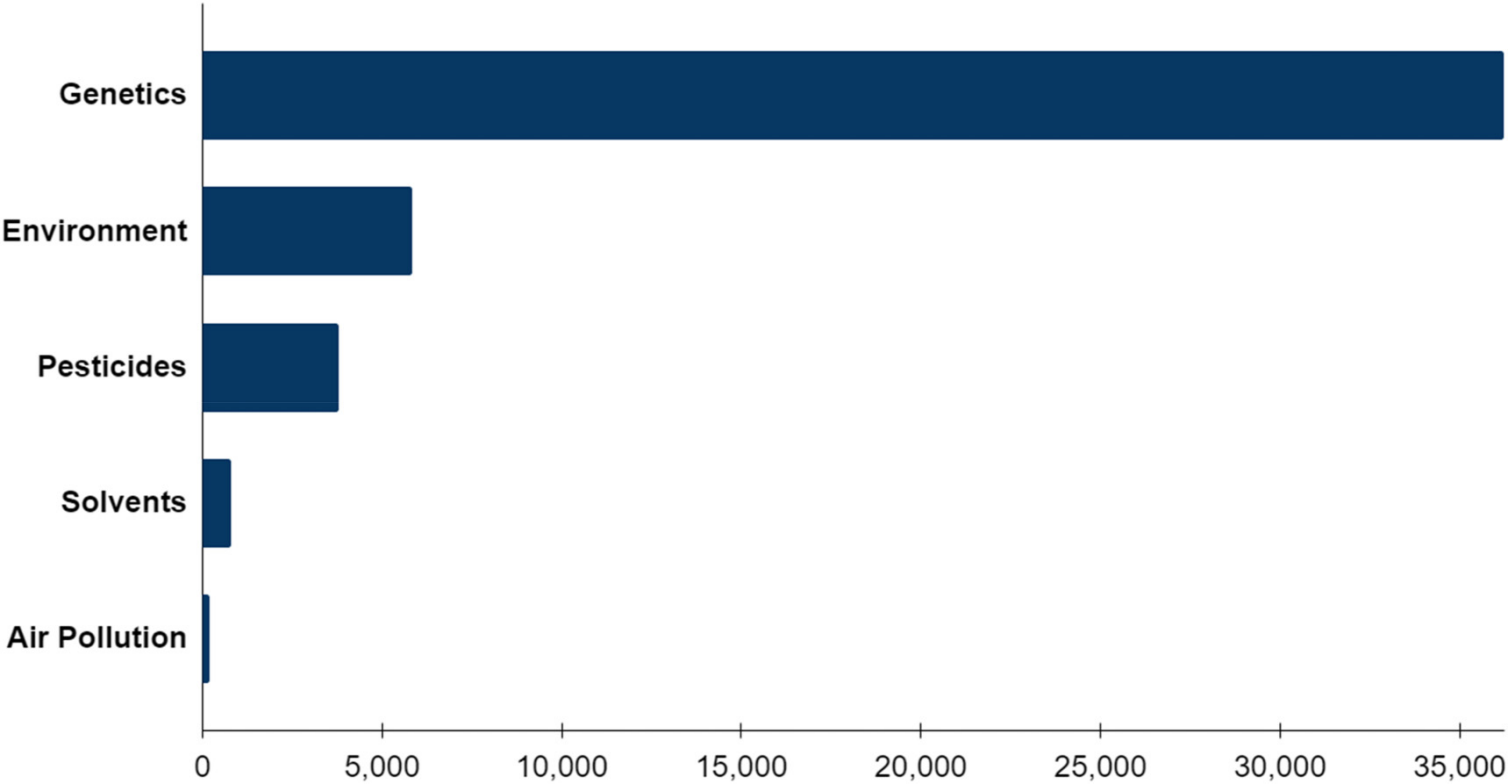
Publications on Parkinson's disease and select topics, 1953–2024*. * Based on a search of PubMed from the U.S. National Library of Medicine with keywords of “*parkinsons* *+* *genetics”, “parkinsons* *+* *environment”, “parkinsons* *+* *pesticides”, “parkinsons* *+* *solvents”, “parkinsons* *+* *air pollution”*.

More funding is, however, not enough. The direction must change (Figure 3). Currently, only about two cents of every Parkinson's research dollar is spent on prevention. In the U.S., which spends more on biomedical research than any other country, almost 60% of the funding comes from pharmaceutical and medical device firms, which are interested in developing new therapies.^134^ Many foundations are focused on a “cure” leaving only a small sliver of public funding aimed at preventing the disease.^135,136^ That must change.

## Goal 3: 100% access to levodopa and care

PD is treatable. Optimal management consists of four pillars: pharmacotherapy; device-aided therapies; multidisciplinary care; and self-management by well-informed patients and caregivers.^114^ Some of these interventions are expensive and may be difficult to spread, but others are very achievable. If 90% of those diagnosed with HIV can receive appropriate (expensive) treatment, why can’t 90 or 100% of those with PD receive appropriate (inexpensive) treatment?

Additional investments should be made to promote self-management strategies. A first step is to offer reliable information tailored to a person's individual needs and preferences, in their own mother tongue. The rapid rise of smartphones globally can enable free access to instruction, including from existing websites that educate people on walking, restoring mobility, and preventing falls. Organizations, such as the Parkinson's Foundation, could make their rich information databases available in as many languages as possible, including versions that can be read on a smartphone. Similarly, the International Parkinson and Movement Disorder Society could expand local and regional training programs for medical professionals.

## A world without Parkinson's disease

In 1961, two Harvard neurologists, Poskanzer and Schwab, postulated that Parkinson's would soon disappear “as a major clinical entity.”^137^ They thought that most PD was a sequela of the sleeping sickness from the early twentieth century. So sure was Poskanzer of his view that he said in *Time* magazine, “I offer a bottle of Scotch to any doctor in the U.S. who can send me a report of a clearly diagnosed case of Parkinson's in a patient born since 1931.”^138^ Poskanzer and Schwab were, of course, wrong as Hoehn and Yahr showed that sleeping sickness accounted for less than 15% of the 802 patients with parkinsonism in their neurology clinic at Columbia University from 1949 to 1964.^139^ In 1967, however, Hoehn and Yahr did not know what caused most of PD.

Today, we do. Genetic factors account for a small proportion of individuals with the disease. And environmental ones, especially chemicals in our food, water, and air, are responsible for the majority. These chemicals have proliferated and spread PD around the world. To stem the tide of Parkinson's requires not a bet, but a plan.

Here we have outlined one to prevent, learn, amplify, and navigate. It is imperfect, incomplete, and ready for improvement, but if implemented, we will slow the rise of the disease, accelerate the development of new therapies, and increase access to levodopa and care. By addressing its root causes and following the bold actions of the HIV community, we can finally set the stage for the disappearance of PD as a major clinical entity.
